# The effects of sampling lateralization on bilateral inferior petrosal sinus sampling and desmopressin stimulation test for pediatric Cushing's disease

**DOI:** 10.1007/s12020-018-1779-x

**Published:** 2018-10-11

**Authors:** Shi Chen, Kang Chen, Lin Lu, Xiaobo Zhang, Anli Tong, Hui Pan, Huijuan Zhu, Zhaolin Lu

**Affiliations:** 1 Department of Endocrinology, Key Laboratory of Endocrinology of National Health Commission, Translation Medicine Centre, Peking Union Medical College Hospital, Peking Union Medical College, Chinese Academy of Medical Sciences, Beijing, China; 2 Eight-year Program of Clinical Medicine, Peking Union Medical College Hospital, Peking Union Medical College, Chinese Academy of Medical Sciences, Beijing, China; 3 Department of Radiology, Peking Union Medical College Hospital, Peking Union Medical College, Chinese Academy of Medical Sciences, Beijing, China

**Keywords:** Cushing’s disease, Pituitary neoplasms, Petrosal sinus sampling, Deamino arginine vasopressin, Pediatrics, Sensitivity

## Abstract

**Purpose:**

Bilateral inferior petrosal sinus sampling (BIPSS) is useful for differential diagnosis of adult Cushing’s disease (CD) but may not be so reliable in pediatric cases. The purpose of this study was to evaluate the sensitivity of BIPSS before and after desmopressin stimulation in pediatric CD, and to explore related factors of false-negative results and meanings of sampling lateralization.

**Methods:**

We retrospectively analyzed 16 pediatric CD patients who underwent 17 BIPSS procedures from 2006 to 2017. CD was diagnosed if inferior petrosal sinus (IPS) to peripheral adrenocorticotropic hormone (ACTH) ratio was >2 at baseline or >3 after desmopressin stimulation. Sampling lateralization was yielded if interpetrosal sinus gradient was >1.4. Magnetic resonance imaging (MRI) was conducted. All the patients underwent surgery and the diagnosis was confirmed.

**Results:**

The sensitivity was 64.7% (11/17) at baseline and 83.3% (10/12) after desmopressin stimulation. After stimulation, BIPSS reached its best sensitivity at 3 min. Sampling lateralization rate was 62.5% and 63.6% before and after stimulation, and the accordant rate with actual tumor lateralization was 50.0% and 42.9%, respectively. The accuracy of MRI in predicting the tumor lateralization was 80.0%. Sampling lateralization rate (81.8% in true-positive, 20.0% in false-negative, *p* = 0.036) and ACTH at dominant IPS (*p* = 0.001) was lower among false-negative patients.

**Conclusions:**

The sensitivity of BIPSS in pediatric CD was low at baseline, but increased after desmopressin stimulation. Sampling lateralization cannot accurately indicate the tumor lateralization, but the absence of sampling lateralization with low ACTH at IPS is a hint of false-negative cases in BIPSS.

## Introduction

Cushing’s syndrome is the result of chronic elevation of glucocorticoids, which may be exogenous or endogenous. Endogenous Cushing’s syndrome can be caused by adrenocorticotropic hormone (ACTH)-dependent and ACTH-independent forms. ACTH-dependent Cushing’s syndrome is caused by excess secretion of ACTH from the pituitary, which is named Cushing’s disease (CD), or by ectopic secretion of ACTH from neuroendocrine tumors, which is named ectopic ACTH syndrome (EAS), or rarely by ectopic secretion of corticotropin-releasing hormone (CRH) [1]. Since many ectopic ACTH-secreting tumors remain occult for a long time, the differential diagnosis of ACTH-dependent Cushing’s syndrome is a challenging issue for clinical management.

Bilateral inferior petrosal sinus sampling (BIPSS) is the gold standard to confirm whether the source of ACTH is pituitary or ectopic secretion. It has been shown that the sensitivity and specificity of BIPSS were extremely high in adult patients [2, 3]. Cushing’s syndrome is relatively rare in children and adolescents with much lower incidence than in adults [4]. Some literatures summarized the experience with CD in children and adolescents, in which BIPSS was generally regarded as a reliable test with comparable accuracy in children and adolescents as in adults [5–7]. Meanwhile, relatively lower sensitivity of BIPSS in children and adolescents was also reported [8], but such observation did not attract much attention. Up to now, there has not been research concerning false negative results of BIPSS before stimulation in children and adolescents, and its reason has not yet been discussed. For the first time in China, this retrospective study in 16 children and adolescents with CD assessed the diagnosis accuracy of BIPSS, and we also innovatively analyzed the related factors of false negative results.

CRH can stimulate the secretion of ACTH from pituitary adenoma, which has been discovered to have the effect of improving the sensitivity of BIPSS in adults [2, 3]. The utilization of CRH stimulation in BIPSS was also validated in children and adolescents [6–8]. As an alternative of CRH, desmopressin has also been used as a stimulant in BIPSS and showed similar sensitivity [9–11]. However, it is unknown whether desmopressin stimulation is as effective in pediatric BIPSS as in adult cases. Here, we describe a series of pediatric BIPSS cases in which desmopressin was used as a stimulant.

Moreover, BIPSS can yield sampling lateralization of ACTH secretion according to the interpetrosal sinus gradient (IPSG), which was supposed to predict the actual lateralization of pituitary adenoma [12, 13]. Here, sampling lateralization was defined as the side whose IPSG to the other side is greater than 1.4 during BIPSS, while the actual tumor lateralization was confirmed by surgery or the imaging. In fact, sampling lateralization by BIPSS was not necessarily consistent with actual tumor lateralization, and in some cases, BIPSS failed to yield explicit sampling lateralization, but its implication remained unclear [10, 14]. In our study, the absence of sampling lateralization was found to have novel clinical significance.

## Patients and methods

### Subjects

This study was approved by the Institutional Review Board of Peking Union Medical College Hospital, Chinese Academy of Medical Sciences. Patient information was obtained with their permission. All the children and adolescents (≤18 years old) admitted to Peking Union Medical College Hospital (PUMCH) who were diagnosed with CD and underwent BIPSS from 2006 to 2017 were included in the study, the total number of which was 16. The diagnosis was based on clinical history, physical examination, serum cortisol and ACTH level, 24 h urine free cortisol (UFC), low- and high-dose dexamethasone suppression test and imaging studies, and was confirmed by transsphenoidal surgery, pathological findings with immunohistochemical staining for ACTH, and clinical or biochemical remission after surgery.

### Imaging

Gadolinium-enhanced magnetic resonance imaging (MRI) and dynamic gadolinium-enhanced MRI of the pituitary gland was conducted in all the patients. A discrete hypointensity in contrast-enhanced imaging or a hypointense lesion in early phase dynamic imaging that became less hypointense with subsequent dynamic cycles suggests the presence of pituitary adenoma. The location of the suspected pituitary adenoma was predicted according to the MRI.

### BIPSS

The BIPSS procedures were performed by experienced radiologists based on the technique described by Doppman et al. [15]. Bilateral femoral vein catheterization was conducted without anticoagulation. 4F catheters were routinely used, and microcatheters were used in patients with narrow inferior petrosal sinus (IPS). Catheters were guided into bilateral inferior petrosal sinuses under the radiographic guidance and retrograde venography was conducted to check the position of catheters. Blood samples were collected from peripheral veins, internal jugular veins as well as left and right inferior petrosal sinuses. All the blood samples were immediately delivered to the laboratory for ACTH assay.

### Desmopressin stimulation test

In the procedure of BIPSS, desmopressin stimulation test was conducted after successful catheterization and sampling at 0 min. Peripheral and inferior petrosal sinus blood samples were simultaneously collected 3, 5, and 10 min after intravenous injection of 10 μg of desmopressin.

### Interpretation of BIPSS and desmopressin stimulation test

An inferior petrosal sinus to peripheral ACTH (IPS:P) ratio of more than 2 before desmopressin stimulation or more than 3 at any time point after stimulation was considered to be consistent with central ACTH secretion [2]. The position of the tumor was predicted to be on one side if the IPSG of that side to the opposite side was greater than 1.4 [2], and such prediction was defined as sampling lateralization. Sampling lateralization before stimulation was considered to be absent if IPSG of both sides was smaller than 1.4.

### Treatment, pathology, and outcome

All the patients underwent transsphenoidal surgery. If no tumor could be visualized, a total pituitary exploration was conducted. The lateralization of the tumor was recorded. Suspected tumor tissue and pituitary tissue adjacent to the tumor were resected for pathological examinations including hematoxylin–eosin staining and immunohistological staining for ACTH. Some patients also underwent pituitary irradiation.

The diagnosis of CD was ultimately confirmed if the pathology demonstrated pituitary adenoma with positive ACTH immunohistological staining. If the pathology result was not available, a clinical diagnosis of CD could be made when the serum cortisol level or 24 h UFC fell back to or below normal range in the follow-up after surgery or irradiation.

Therapeutic outcome was classified into three types: remission, partial remission, and non-remission. Patients were categorized as “remission” if the serum cortisol was below 5 µg/dl after treatment, and as “partial remission” if the serum cortisol or 24 h UFC was lower than preoperative level and was within normal range.

### Statistical analysis

Continuous variables are presented as average ± standard deviation or median (first quantile, third quantile) and qualitative variables as count and frequency. Normality of distributions was tested with Shapiro–Wilk test. Continuous data was analyzed with *t*-test or Mann–Whitney *U* test and categorical data with Fisher’s exact test. Correlation between continuous data was analyzed with Spearman analysis. *p*-Value <0.05 was considered statistically significant.

## Results

### Baseline characteristics

Data were available in 17 BIPSS procedures performed in 16 patients. Catheterization to bilateral inferior petrosal sinuses was successfully undertaken in all the patients. No severe adverse effect occurred. Among the 16 patients, 6 (37.5%) were male and 10 (62.5%) were female, whose mean age was 15.5 ± 2.9 years (range 9.8–18.7 years). The average height was 152.5 ± 13.7 cm (−6.3 to 1.2 SDS) and average weight was 65.0 ± 17.2 kg (−0.9 to 2.9 SDS). The median duration of disease was 2.3 (1.2, 3.1) years (range 0.5–9.0 years). The average ACTH level was 56.6 ± 23.2 ng/L, and median 24 h UFC level was 495.0 (233.8, 654.4) μg. Detailed baseline characteristics of each patient are shown in Table 1.

**Table 1:** Baseline characteristics of 16 children and adolescents with Cushing’s disease

No.	Sex	Age	Duration of disease (years)	Height (cm)	Height SDS	Weight (kg)	Weight SDS	ACTH (ng/L)	24 h UFC (μg)
1	F	15.2	6.0	146	−2.6	57.5	0.9	56.0	373.0
2	F	14.3	4.0	144	−2.5	42.5	−0.8	14.9	453.9
3	M	9.8	0.5	145	0.3	67.5	2.9	47.7	1144.7
4	M	13.3	1.0	156	−1.0	63.0	1.0	50.9	649.7
5	F	14.6	3.0	140	−3.5	57.5	1.0	44.7	111.6
6	M	16.2	3.0	175	0.5	115.0	2.0	67.8	137.6
7	F	15.4	3.0	153	−1.4	58.0	2.0	46.6	668.5
8	F	18.6	0.7	161	0.1	70.0	2.2	65.4	562.4
9	F	17.7	0.5	159	−0.4	73.0	2.4	105.0	2512.8
10	F	16.7	1.5	167	1.2	76.0	2.7	93.5	1183.5
11	M	11.4	2.0	139	−1.4	44.5	0.4	34.0	596.8
12	F	17.2	9.0	126	−6.3	45.5	−0.9	62.2	246.2
13	M	16.7	2.5	168	−0.7	80.0	1.7	79.0	69.1
14	M	13.5	1.5	135	−3.7	60.0	0.8	53.9	196.8
15	F	18.7	3.5	161	0.0	63.0	1.4	59.3	488.6
16	F	17.2	1.3	165	0.9	67.0	1.9	24.5	501.5

### Sensitivity of BIPSS before or after desmopressin stimulation in diagnosis of Cushing’s disease

Before desmopressin stimulation, 11 of 17 (64.7%) procedures had a maximal IPS:P ratio of 2 or greater. One of the patients (No. 16) underwent two BIPSS procedures in 1 month, both of which had a maximal IPS:P ratio of less than 2.

Desmopressin stimulation was conducted in 12 procedures of 11 patients, and increased the maximal IPS:P ratio in 9 procedures. After stimulation, 10 (83.3%) procedures had a peak IPS:P ratio of 3 or greater. Among the 6 false negative procedures before desmopressin stimulation, 4 procedures (3 patients) underwent stimulation, 2 of which had a peak IPS:P ratio of 3 or greater. Maximal IPS:P ratio of the patient No. 16 increased after stimulation in the two procedures, but did not reach 3, who is the only false negative patient after desmopressin stimulation. The detail of IPS:P ratio before and after desmopressin is demonstrated in Table 2.

**Table 2:** Tumor lateralization, details of BIPSS, and treatment outcome of 16 children and adolescents with Cushing’s disease

No.	Lateralization				ACTH at dominant IPS	IPS:P before stimulation	IPS:P after stimulation	Treatment outcome
	By BIPSS without desmopressin stimulation	By BIPSS with desmopressin stimulation	By MRI	By surgery				
1	No	N.A.	Left cavernous sinus	Not found	57.7	1.1	N.A.	Partial remission after radiotherapy
2	R	N.A.	Both	Both	56.38	5.82	N.A.	Partial remission
3	L	L	Not seen	Middle	>1250	16.19	15.01	Remission
4	L	L	L	L	1225	20.52	26.94	Partial remission
5	L	N.A.	L	L	1201	8.77	N.A.	Remission
6	No	no	R	R	>1250	17.34	9.12	Remission
7	No	N.A.	Middle	Middle	55.3	1.61	N.A.	Remission
8	R	N.A.	R	R	>1250	17.22	N.A.	Remission
9	R	R	Middle	L	723	11.61	7.76	Remission
10	R	R	R	L	745	5.48	8.28	Remission
11	L	L	L	Both	169	5.91	22.2	Remission
12	No	Contradictory	L	L	359	6.24	11.27	Non-remission
13	L	L	L	L	685	11.12	28.54	Partial remission
14	No	R	R	R	81.1	1.77	4.8	Non-remission
15	R	Contradictory	Not seen	R	48.8	1.53	18.97	Non-remission
16	No	No	R	R	23.1 40.4	1.07 1.08	1.34 2.05	Remission

### IPS:P ratio at different time points

**Fig. 1:**
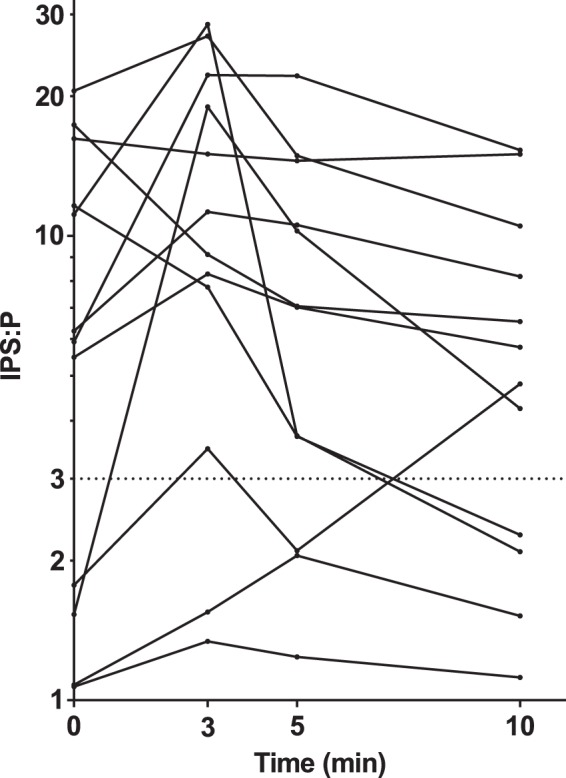
Maximal inferior petrosal sinus to peripheral (IPS:P) ACTH ratio at different time points in 12 BIPSS procedures with desmopressin stimulation in 11 children and adolescents with Cushing’s disease

As demonstrated in Fig. 1, in 10 of the 12 procedures, the IPS:P ratio reached its peak before desmopressin stimulation or at 3 min. The patients whose IPS:P ratio neither reached 2 before desmopressin stimulation nor reached 3 at 3 min would not had a maximal IPS:P ratio greater than 3 at 5 or 10 min in the procedure. Among the 12 procedures with desmopressin stimulation, 10 (83.3%) had a maximal IPS:P ratio that was greater than 2 at 0 min or greater than 3 at 3 min.

### Sampling lateralization, MRI, and surgery findings

Tumor lateralization was confirmed during surgery in 15 patients, 6 (40.0%) had a tumor located on the left side of the pituitary, 5 (33.3%) on the right side, 2 (13.3%) on both sides, and 2 (13.3%) in the middle. The concordance of tumor lateralization by BIPSS, MRI, and surgery is demonstrated in Table 2. After the surgery, 9 of the 16 patients were in remission and 3 patients were in partial remission. In patient No. 1, the tumor invaded left cavernous sinus, and was not resected in surgery, but partial remission was achieved after subsequent sellar radiotherapy. The other 3 patients were not in remission. In all the patients except No. 1, CD was confirmed by pathological results. The treatment outcome of each patient is listed in Table 2.

Suggestive MRI was found in 14 of the 16 patients (87.5%). Tumor was lateralized to the left or the right side by MRI in 10 of the 16 patients (62.5%), 5 were lateralized to the left, 4 of which were consistent with operation finding, and 5 were lateralized to the right, 4 of which were consistent with operation finding. In total, lateralization by MRI was in concordance with operation finding in 8 (80.0%) patients. Tumor was observed to locate on both sides of pituitary by MRI in 1 patient, which was confirmed by surgery. MRI also indicated midline tumor in 2 patients, one of which was proved to be correct by surgery. Besides, 2 patients had negative MRI finding.

BIPSS yielded an IPSG above 1.4 in 10 of the 16 patients before desmopressin stimulation, thus the sampling lateralization rate was 62.5%. Operation findings confirmed tumor lateralization in 7 patients, while tumor was found to be located in the middle or occupy both sides in the other 3 patients. In 5 patients, sampling lateralization before stimulation correctly predicted the actual tumor lateralization, the accordant rate of which was 50.0%. In the 6 patients without sampling lateralization, only 1 (16.7%) patient was proved to have a midline tumor, while tumor was found by operation on one side of the pituitary in 4 patients, and tumor was not found by surgery in 1 patient. After desmopressin stimulation, sampling lateralization rate was 63.6% (7/11), and the accordant rate of sampling lateralization and actual tumor lateralization was 42.9% (3/7). In the other 4 patients, 2 had IPSG below 1.4 at all the time points and 2 had contradictory results at different time points, but operation confirmed that the tumor was on one side in all these patients.

### Related factors of diagnosis accuracy

**Fig. 2:**
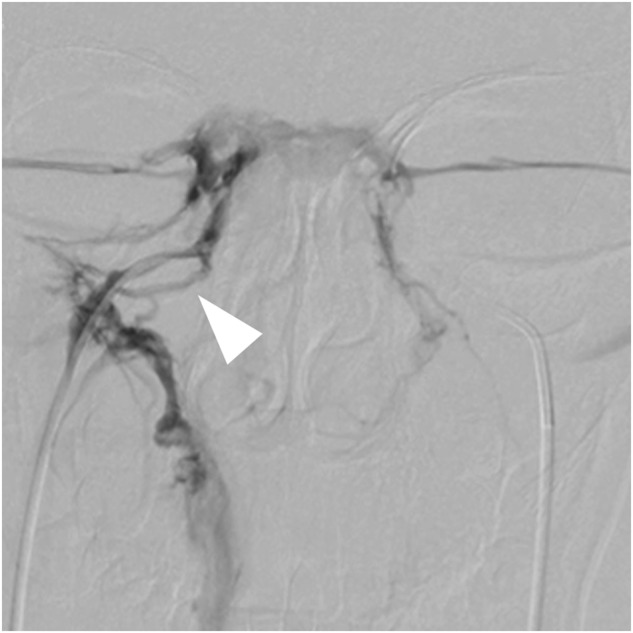
The angiography of inferior petrosal sinus in a true positive patient. The arrowhead indicates the plexiform IPS

Clinical characteristics were compared between 11 true positive and 5 false negative patients (Table 3). The differences of age, duration of disease, height, height SDS, weight, weight SDS, BMI, ACTH level, 24 h UFC, sex, and proportion of patients with tumor on the right side between the two groups of patients were not significant. Images of angiography were also examined, and plexiform IPS was found in some true positive patients (Fig. 2), while no anatomical variation of IPS was detected among the false negative cases. However, sampling lateralization rate before stimulation was 20.0% (1/5) among false negative patients and 81.8% (9/11) among true positive patients, the difference of which was significant (*p* = 0.036). The absence of sampling lateralization was related to false negative cases. Besides, baseline IPS:P ratio was positively correlated with the baseline ACTH concentration at the dominant IPS (*ρ* = 0.909, *p* < 0.001), and the baseline ACTH concentration at the dominant IPS was significantly lower (*p* = 0.001) among the false negative patients. In addition, true positive patients tended to had a higher frequency of actual tumor position on the left side (*p* = 0.093).

**Table 3:** Clinical characteristics of false negative and true positive patients in BIPSS

	False negative (*n* = 5)	True positive (*n* = 11)	*p*-Value
Age (years)	16.1 ± 2.0	15.2 ± 2.8	0.532
Duration of disease (years)	3.0 (1.5, 3.5)	2.0 (0.9, 3.0)	0.377
Height (cm)	152.0 ± 12.0	152.7 ± 15.0	0.930
Height SDS	−1.4 ± 1.9	−1.2 ± 2.2	0.920
Weight (kg)	61.1 ± 3.9	66.8 ± 20.7	0.560
Weight SDS	1.2 ± 0.5	1.3 ± 1.3	0.780
BMI	26.7 ± 3.6	28.1 ± 4.5	0.564
ACTH (ng/L)	48.1 ± 14.0	60.5 ± 26.0	0.339
24 h UFC (μg)	488.6 (373.0, 501.5)	562.4 (191.9, 897.2)	0.827
ACTH at dominant IPS (ng/L)	52.1 (42.5, 57.1)	745.0 (522.0, 1237.5)	0.001*
Tumor size (mm)	4.8 ± 0.5	7.3 ± 4.3	0.267
Sex			0.588
Male	1	5	
Female	4	6	
Duration of disease			0.245
≤1 year	0	4	
>1 year	5	7	
Sampling lateralization before stimulation			0.036*
Presence	1	9	
Absence	4	2	
Actual position			0.093
Left	0	6	
Others	5	5	
Actual position			0.245
Right	3	2	
Others	2	9	

* p<0.05

## Discussion

Here we reported the first series of children and adolescents with CD who underwent BIPSS in China. As one of the largest series that systematically reported the use of desmopressin in BIPSS for pediatric CD, this study validated that desmopressin stimulation can increase the sensitivity of BIPSS in children and adolescents. Thus, desmopressin can be an effective and easily available alternative of CRH in pediatric BIPSS. This is also the first study that focused on the reliability of BIPSS in children and adolescents and thoroughly analyzed the characteristics of false negative cases. The most prominent finding of our study is that BIPSS is not as accurate as reported previously for diagnosis and tumor lateralization in children and adolescents with CD, even in experienced hands, and we suggest that the absence of sampling lateralization before stimulation together with low ACTH level at IPS is a potential sign of false negative result in BIPSS.

### Sensitivity of BIPSS and the effect of desmopressin stimulation

In this study, the sensitivity of BIPSS without desmopressin stimulation for the diagnosis of CD in children and adolescents was only 64.7%, while the sensitivity with desmopressin stimulation was 83.3%. In most of the previous studies whose subjects were mainly adults, the sensitivity of inferior petrosal sinus sampling (IPSS) for diagnosis of CD was more than 80% at baseline and more than 85% after stimulation [3], both of which were higher than our result. Since CD mainly affects adults, there were few studies about IPSS in children and adolescents, as summarized in Table 4. The sensitivity of IPSS at baseline in our study was lower than previous publications reporting sensitivities from 75.9 to 100% in children and adolescents [6–8, 16]. While the study by Batista et al. [6] and by Magiakou et al. [7] reported sensitivity of over 90%, the study by Storr et al. [8] reported a much lower sensitivity of 75.9%. However, we noticed that in the study by Storr et al. [8], the success rate of sampling was relatively low and the sensitivity of BIPSS before stimulation was even lower in adults than in pediatric patients, which implied that the low sensitivity in this study may be partly due to the lack of experience. In PUMCH, BIPSS has been used as a conventional technique for diagnosis of CD in adults, with a sensitivity of 90.1% before desmopressin stimulation and 95.6–98.9% after stimulation, as reported previously [13, 17]. Besides, the success rate of catheterization was high in our series. Thus, it was improbable that such a high false negative rate in our series was caused by technical problems. As a result, our data suggests that the phenomenon of lower reliability of BIPSS in children and adolescents actually exists.

**Table 4:** Summary of literatures about the diagnosis of Cushing’s and tumor lateralization by IPSS in children and adolescents [5–8, 16, 18, 19]

District	Author	Age	Sensitivity		Sampling lateralization rate	Accordant rate of lateralization
			Baseline	Stimulation^a^		
China	Our study	15.5 ± 2.9 (9.8–18.7)	64.7% (11/17)	83.3% (10/12)	62.5% (10/16) (baseline) 63.6% (7/11) (stimulation)	50.0% (5/10) (baseline) 42.9% (3/7) (stimulation)
US	Lonser et al.^b^	5.8–20.8	N.A.	99.3% (139/140)	87.9% (123/140)	81.8% (57/82)
	Batista et al.	13 ± 3.2 (5.3–18.7)	90.2% (83/92)	96.7% (88/91)	84.1% (58/69)	60.3%(35/58)
	Magiakou et al.	14 ± 4	95.3% (41/43)	97.7% (42/43)	N.A.	67% (baseline) 76% (stimulation)
UK	Storr et al.	12.3 ± 3.5 (5.7–17.8)	75.9% (22/29)	86.2% (25/29)	75.8% (25/33^c^)	81.8%(27/33)
	Joshi et al.	13.4 (6.6–17.8)	N.A.	100% (19/19)	73.7% (14/19)	100% (14/14)
	Lienhardt et al.	10.7–18.8	N.A.	100% (7/7)	85.7% (6/7)	100% (7/7)
India	Shah et al.	14.9 ± 2.5	88.9% (8/9)	N.A.	N.A.	N.A.

^a^CRH was used in all these previous studies with stimulated BIPSS, and desmopressin was used in our study

^b^Part of the cases have been reported by Magiakou et al.

^c^Samples obtained from high internal jugular veins in 4 patients

After desmopressin stimulation, the sensitivity of BIPSS jumped to 83.3%. Similarly, such an increase was also observed in previous literatures reporting sensitivities after CRH stimulation of 86.2–100% [5–8, 18, 19]. It should be noticed that CRH was used as a stimulant in all these studies in children and adolescents, but in our study, desmopressin was used instead, since CRH is not approved by China Food and Drug Administration for clinical application in China. In limited studies reporting BIPSS with desmopressin stimulation, the sensitivity after stimulation ranged from 92.1 to 97.2% [9–11]. Compared with these studies whose subjects were mainly adults, the sensitivity of BIPSS after desmopressin stimulation in our pediatric series was slightly lower. Nevertheless, our study demonstrated that desmopressin stimulation can increase the IPS:P ratio in most of the children and adolescents, and thus increase the sensitivity of BIPSS. Thus, stimulation of CRH or desmopressin is necessary to maximize the sensitivity of BIPSS. Although desmopressin is a hemostatic agent and may increase thromboembolic risks [20], there was no evidence of severe adverse effect when desmopressin was used in pediatric cases in our study. As a result, desmopressin stimulation is safe and effective in BIPSS for children and adolescents with CD.

In our series, BIPSS reached its best sensitivity at 3 min, and sampling at 5 and 10 min did not improve the sensitivity. Latency to the peak IPS:P ratio after stimulation was lower in our data than previous studies that employed CRH stimulation, in which the latency was 5–25 min [21, 22]. It can be speculated that sampling at 3 min after desmopressin stimulation is enough for a correct diagnosis, which can reduce the duration of BIPSS, and thus enhance the tolerance of children and adolescents to this procedure.

### False negative results of BIPSS

Although BIPSS has been thoroughly studied in adults, the causes of false negative results in children and adolescents have not yet been discussed. Here, we tried to investigate the cause of false negative BIPSS in children and adolescents.

Several potential causes of false negative results in IPSS have been summarized in previous studies. Anatomical variations of IPS were reported to influence the venous drainage of pituitary or preclude the correct positioning of catheter, which might lead to erroneous results of IPSS [23, 24]. Such variations are common, as reported previously that in 1/4 of the cases the IPS was plexiform [25], but in most cases, they did not lead to diagnostic error [23, 26]. Similarly, in our series, IPS of less common types was not related to false negative results. In addition, the measuring error of ACTH has also been proposed to be a potential reason for false negative results, but in our cases, the IPS:P ratios were not near the threshold, which necessarily precluded this possibility. As a result, possible causes reported in previous studies failed to explain such a high false negative rate in our study.

After further analysis of our data, the cause of false negative cases of BIPSS remained unclear, but it is noteworthy that the sampling lateralization rate at baseline was significantly lower among false negative patients than true positive patients. The absence of sampling lateralization indicates that the drainage of ACTH-containing blood is bilateral or instable, which may dilute the ACTH in IPS. Moreover, it can be noticed that false negative patients tended to have lower ACTH and smaller tumor size, which implied that the amount of ACTH secreted by the pituitary tumor might be smaller in these patients. Although such observation did not reach the level of significance, it may influence the ACTH level of IPS in combination with the bilateral drainage. Thus, the absence of sampling lateralization before stimulation in patients with IPS:P ratios below threshold should raise the suspicion for false negative results. Although stimulation may increase the secretion of ACTH to maintain its high concentration despite the bilateral drainage, the absence of sampling lateralization before stimulation may be related to the false negative results after stimulation. Therefore, sampling lateralization should be taken into consideration when interpreting the result of BIPSS.

It was reported that IPS:P ratio before or after stimulation was positively associated with the ACTH concentration at the dominant IPS [27]. As a result, low ACTH level at dominant IPS might help identify false negative cases in BIPSS [12]. In accordance with these, our study also found that baseline ACTH level at dominant IPS was relatively lower among false negative patients. Low ACTH at IPS may suggest the presence of these factors that can affect the reliability of BIPSS, such as improper catheterization or anatomical variations that dilute the ACTH at IPS.

The absence of sampling lateralization and low ACTH level at IPS were two unique characteristics of false negative cases in BIPSS. However, it is unclear whether they can distinguish false negative and true negative patients, and further studies are needed to find more reliable index for identification of false negative cases. Methods that can improve the sensitivity of BIPSS to reduce false negative cases, such as desmopressin stimulation, are still of great importance.

Otherwise, no significant difference of clinical characteristics was observed between true positive and false negative patients. The significant results above were not validated by more rigorous statistical tests such as multi-variable analysis or Bonferroni corrections due to the limited number of subjects, which may overestimate their effect. Further studies in larger cohorts are needed to confirm these observations.

### Sampling lateralization and actual tumor lateralization

Sampling lateralization rate by BIPSS without desmopressin stimulation was only 62.5% in our study, and the accordant rate with actual tumor lateralization was only 50%. Desmopressin stimulation not only failed to improve the efficacy and accuracy of tumor lateralization, but also led to contradictory results. Such negative effect of stimulation on tumor lateralization has also been suggested by other study [6]. Therefore, the clinical usefulness of BIPSS for tumor lateralization in our cases was limited. As reported by previous studies in children and adolescents (Table 4), sampling lateralization rates by BIPSS ranged from 73.7 to 100%, and their accordant rates with actual tumor lateralization were 58.7–100% [5–8, 16, 18, 19], both of which were higher than our data. It should be noticed that the criteria of calculating the tumor lateralization accuracy in these studies were slightly different. For example, in the study of Lonser et al. [5], only those patients whose tumor was of the midline were included in the calculation of lateralization accuracy, while patients with a midline tumor were also included in the calculation of Storr et al. [8].

**Fig. 3:**
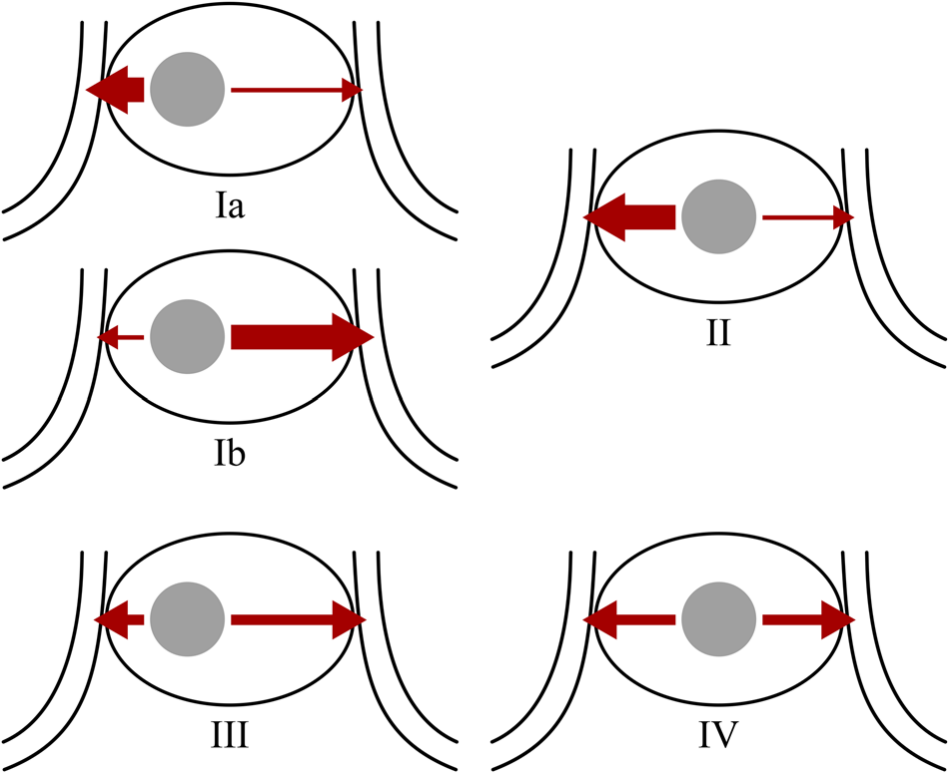
Schematic of the four types of relationship between lateralization based on the sampling lateralization and actual tumor lateralization. The large ellipse represents a pituitary and the small grey circle represents an ACTH-secreting adenoma. Red arrows indicate the venous drainage of the adenoma

Here, we should emphasize that the sampling lateralization and the actual tumor lateralization are two different concepts. Sampling lateralization characterizes the dominant venous drainage of tumor, while the tumor lateralization confirmed by the operation is determined by the geometric position of the tumor. The relationship of sampling lateralization and tumor lateralization can be divided into four types (Fig. 3). In type I lateralization, venous blood from the tumor robustly drains to one side, so sampling lateralization can be detected, and tumor is also located on one side of the pituitary. This type can be further divided into two subtypes. Sampling lateralization and actual tumor lateralization is accordant in type Ia but is discordant in type Ib. In type II lateralization, the venous drainage of a midline tumor is asymmetrical, which leads to sampling lateralization. In type III lateralization, tumor is located on one side of the pituitary, but the venous blood drains equally to both sides, which leads to the absence of sampling lateralization. In type IV lateralization, the venous blood from a midline tumor drains symmetrically to both sides, so sampling lateralization cannot be detected. All these types of lateralization were observed in our data, and we discovered that type III and type IV were related to false negative result of BIPSS in the diagnosis of CD. The possible mechanism is that these two types of lateralization lead to dilution of the ACTH from pituitary tumor, as discussed above. Moreover, the ACTH level at dominant IPS is lower in type III or type IV lateralization than that in type I and type II lateralization, provided that the total amount of ACTH produced by the pituitary is identical. This may partly explain the association between low ACTH level at IPS and false negative results as described above.

In addition, MRI may also help to predict the position of pituitary tumor. The lateralization accuracy of MRI was also unsatisfactory in our series though slightly higher than that of BIPSS. However, MRI detected pituitary adenoma with high sensitivity in our series, which is higher than that of unstimulated BIPSS. Nevertheless, such imaging study is unable to distinguish ACTH-secreting tumors from non-functional ones, thus BIPSS still has an irreplaceable role in the confirmation of CD.

### Clinical implications

With the ability to confirm the central secretion of ACTH, BIPSS is an important investigation to distinguish CD and EAS. In adults, CD accounts for 60–70% of Cushing’s syndrome while EAS 5–10% [1]. The proportion of CD in children and adolescents is similar to that in adults, but EAS in children and adolescents is rare, which accounts for approximately 1% [4]. That is, the ratio of CD to EAS is higher in children and adolescents than in adults. Considering the lower sensitivity of BIPSS, especially among the patients with type III or type IV lateralization, the negative predictive value of BIPSS is unsatisfactory in children and adolescents, which means that the lack of a central to peripheral gradient in BIPSS cannot exclude EAS. Desmopressin stimulation can increase the sensitivity, but the negative predictive value is still not high enough. On the contrary, positive predictive value of BIPSS is high due to its specificity of nearly 100% [7, 28]. As a result, in children and adolescents, BIPSS should only be used for definite diagnosis of CD, rather than exclusion of EAS, nor lateralization of pituitary adenoma.
